# “Assessing exposure of printing factory workers in thailand to selected heavy metals using urine and hair as non-invasive matrices”

**DOI:** 10.1186/s12889-022-14807-0

**Published:** 2023-01-05

**Authors:** Patthrarawalai Sirinara, Yupin Patarapongsant, Siwaporn Nilyai, Kanidta Sooklert, Thasinas Dissayabutra, Rojrit Rojanathanes, Amornpun Sereemaspun

**Affiliations:** 1 Department of Preventive and Social Medicine, King Chulalongkorn Memorial Hospital, Bangkok, Thailand; 2 Department of Preventive and Social Medicine, Faculty of Medicine, Chulalongkorn University, Bangkok, Thailand; 3 Behavioral Research and Informatics in Social Sciences Research Unit (RU-BRI), SASIN School of Management, Chulalongkorn University, Bangkok, Thailand; 4 Center of Excellence in Nanomedicine, Department of Anatomy, Faculty of Medicine, Chulalongkorn University, Bangkok, Thailand; 5 Department of Biochemistry Metabolic Disease in Gastrointestinal and Urinary System Research Unit, Faculty of Medicine, Chulalongkorn University, Bangkok, Thailand; 6 Department of Chemistry, Faculty of Science, Chulalongkorn University, Bangkok, Thailand

**Keywords:** Heavy metals, Printing factory workers, Urine, Hair, Thailand

## Abstract

**Background:**

There are few thorough studies on the extent and inter-element relationships of heavy metal contamination in printing factory workers, especially in developing countries. The objective of this study was to determine the levels of eight heavy metals, including arsenic (As), cadmium (Cd), chromium (Cr), nickel (Ni), cobalt (Co), lead (Pb), mercury (Hg), and manganese (Mn), in urine and scalp hair of printing industry workers, and assess inter-element correlations.

**Methods:**

We examined a total of 85 urine samples and 85 scalp hair samples (3 cm hair segments taken from near the scalp) in 85 printing workers from a printing house in Bangkok, Thailand. We used an interviewer-administered questionnaire about participants’ printing techniques, work characteristics, and work environment. Urine and scalp hair samples were analyzed for levels of each element using the inductively coupled plasma optical emission spectrometry (ICP-OES) technique.

**Results:**

As, Cd, Cr, Ni, Pb were detected in urine with the geometric mean concentration range of 0.0028–0.0209 mg/L, and Hg, Pb, Ni, Cd, Co, Mn, Cr were detected in hair samples (0.4453–7.165 mg/kg dry weight) of printing workers. The geometric mean Ni level was significantly higher in the urine of production line workers than back-office personnel (0.0218 mg/L vs. 0.0132 mg/L; *p* = 0.0124). The other elements did not differ significantly between production line and back-office workers in either urine or hair. There was also a strong, statistically significant positive correlation between Ni and Co levels in hair samples of workers (*r* = 0.944, *p* < 0.0001).

**Conclusions:**

Average concentrations of most of the metals in urine and hair of printing workers were found to be above the upper reference values. The significantly higher concentrations of Ni in production line workers might be due to more exposure to printed materials. A strong inter-element correlation between Ni and Co in hair samples can increase stronger health effects and should be further investigated. This study reveals possible dependencies and impact interactions of heavy metal exposure in printing factory workers.

**Supplementary Information:**

The online version contains supplementary material available at 10.1186/s12889-022-14807-0.

## Background

Heavy metals are elements with a high atomic weight and a density of at least five times that of water [1]. The main ones are arsenic (As), mercury (Hg), lead (Pb), nickel (Ni), cadmium (Cd), cobalt (Co), manganese (Mn), and chromium (Cr). These are all toxic metals and have shown detrimental health effects in humans. As, Ni, Cd, and Cr are Group 1 human carcinogens according to the International Agency for Research on Cancer (IARC) [2]. Ni, Co, Mn, and Cr can irritate the respiratory tract and impair lung function [3–5]. As, Hg, Pb, Cd, and Mn are nephrotoxic metals [6–9].

Heavy metal concentrations in industrial environments are far higher than in natural settings [10–12]. The printing industry poses occupational health challenges because its workers are exposed to a range of metal elements through inhalation, skin contact, or ingestion [13, 14]. Printing technologies are classified as digital (ink jet), offset lithographic, screen, flexographic, gravure, or 3D printing [13]. Digital (ink jet) and offset lithography are two of the most widely used printing methods in large-scale printing factories [13, 15, 16]. Offset lithography is a traditional printing method that uses metallic plates to transfer ink onto a rubber sheet. The image is then rolled onto the printing surface [17]. Rather than using metallic plates and rubber blankets to transfer an image, digital (ink jet) printing involves the direct placement of liquid ink onto the printing surface [15, 17]. Each printing process consists of three main steps: prepress, which is the conversion of a printed image into an image carrier; printing operations; and assembly of printed materials [17]. Printing colorants include a diverse array of metal nanoparticles such as Pb, Cd, Cr (VI), Ni, Silver (Ag), Copper (Cu) complexes, and metallo-organic compounds [18–22].

Several studies have reported heavy metal contamination from the printing industry. In China, a recent research pointed out the risk of high heavy metal exposure in industrial sewage sludge from printing industry as a particular environmental health issue [23]. A recent study in India demonstrated that a dominant contribution of sources to the heavy metals in the air was from printing factory [24]. Increasing studies have represented that metal particles are emitted from printers [25, 26]. Workers inhale those emissions of printers during routine work may experience health issues. Biological monitoring is an integral part of the occupational health and safety strategy to address heavy metal exposure in printing workers. Previous studies have demonstrated internal doses of heavy metal residues, their metabolites, or markers of subsequent health effects in blood, urine, and hair of printing workers [27–31]. Within the occupational context, biological monitoring helps with accurate assessment of worker exposure, especially where environmental monitoring alone may underestimate the exposure risk [32, 33].

There are around 100 printing houses in Bangkok, Thailand, most of which are small-to-medium-scale. Large- scale establishments, being defined as more than 75 workers, account for less than 1% of the total number. Although many heavy metals are recognized to be highly toxicants, to date, monitoring of this exposure in the printing industry is limited. The authors selected a large-scale printing house accessible at the time of the study to assess the health burden and potential metal exposures as a critical first step in gauging the public health burden of metal exposure and in guiding recommendations to promote health of workers in the factory. This study aimed to assess eight heavy metals (As, Hg, Pb, Ni, Cd, Co, Mn, and Cr) and their inter-element relationships in printing factory workers via non-invasive approaches such as urine and scalp hair samples.

## Methods

### Study area and study participants

**Fig. 1:**
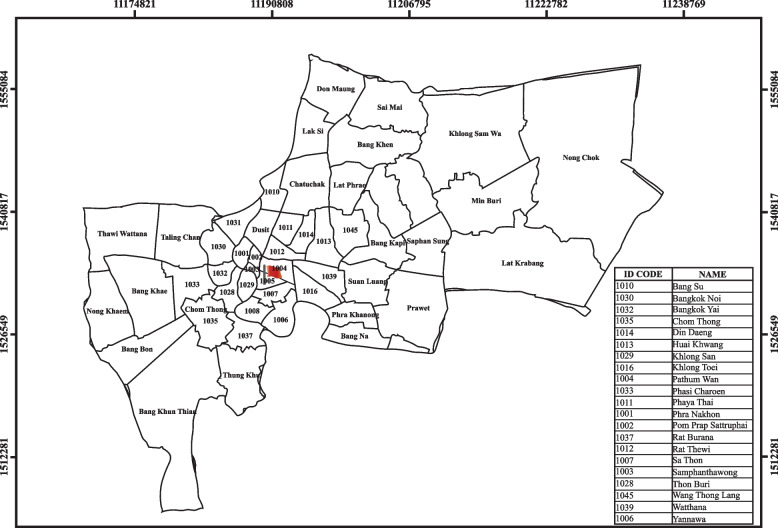
Geographic map of the printing factory and the surrounding area in Bangkok, Thailand

The study was conducted at a large-scale printing house in Pathum Wan district, Bangkok, Thailand. Pathum Wan district is located in the central part of Bangkok and has an area of around 1.95 square kilometers with a mixed educational, commercial, and residential area (Fig. 1). The Printing House has 103 employees and two divisions: the printing division and the back-office division. It uses digital (ink jet) printing methods for on-demand printing and quick turnaround times, and offset lithographic printing methods for a high-volume production line. At the time of the study, the printing house operated Monday to Friday, with work usually scheduled for 8AM–5PM, and workers all doing one eight-hour shift per day. This study enrolled printing workers with records of their work characteristics and workplace environment. The inclusion criteria were workers aged 20–60 years who worked in one of the printing factory divisions: the printing division, and the back-office division. We excluded anyone who was pregnant or who refused to participate in the study. The study included an interviewer-administered questionnaire and the collection of urine and scalp hair samples. Permission to conduct the study was obtained from the factory owners. All study participants provided signed informed consent.

The questionnaire collected information on gender, age, work commute mode (by personal car, motorcycle, or public transport), work characteristics (printing or back-office), printing techniques, and years of employment. Environmental information included air ventilation and type (general or local ventilation), and behavioral information included smoking cigarettes and drinking alcohol. Urine and hair samples were collected from eligible participants. They were sent for analysis of levels of As, Hg, Pb, Ni, Cd, Co, Mn, and Cr using the inductively coupled plasma optical emission spectrometry (ICP-OES) technique. This study was approved by the Ethics Committee of the Institutional Review Board of the Faculty of Medicine, Chulalongkorn University (IRB No. 2016–558/59), and the principles of the Declaration of Helsinki were followed. Participant flow is shown in Fig S1.

### Sample collection and preparation

Urine samples were collected at the end of the last shift at the end of a working week. In total, 5 mL to 20 mL of each participant’s urine was collected in a sterile nitric acid-washed polyethylene container. Samples were acidified and diluted with 2% HNO_3_ in a 1:1 dilution ratio within 48 h of collection. The samples were stored at 4 °C and analyzed within two weeks (modified from Burden et al., 1998) [34].

Hair samples were collected by cutting a 3 cm hair segment from near the scalp of each participant. The quantity of each participant’s hair sample required for analysis was 20 mg. The hair samples were stored at room temperature in nitric acid pre-washed polyethylene containers. Once in the laboratory, each sample was cut into smaller pieces and washed with MilliQ water. The washed samples were dried at 40 °C overnight in the oven and then weighed. The washed hair samples were added to glass beakers containing 5 mL of strong nitric acid, digested and then heated to 150 °C until the samples were homogenized into solutions. After digestion, 1 mL of H_2_O_2_ was added to the solution. The solutions were subsequently diluted to 50 mL using MilliQ water (modified from Ming-Jin He et al., 2016) [35].

### Quantification of the elements

The concentration of all elements in both urine and hair samples was measured using inductively coupled plasma optical emission spectrometry (ICP-OES) instruments (Optima 2100 DV, PerkinElmer, USA). The instrument was calibrated using solutions of multi-element standards (Multi-Element Calibration Standard 3, PerkinElmer), and calibration curves were made from five different concentrations (0.01, 0.05, 0.1, 0.5 and 1 mg/L) of this standard solution. The instrument's working parameters were: plasma flow 15 L/min; nebulizing flow 0.8 L/min; and radiofrequency power 1300 W. The following wavelengths (nm) were used to measure each element's emission intensity: 193.696 As; 194.168 Hg; 220.353 Pb; 221.648 Ni; 228.802 Cd; 238.892 Co; 257.610 Mn; and 267.716 Cr. The solution of HNO_3_ and H_2_O_2_ was diluted to the same concentration as in the sample preparation step, to use as a blank sample. The blank sample was analyzed and subtracted from each measurement.

### Quality assurance and quality control

The validity of the analytical procedure was examined by certified reference material of Human hair ERM®-DB001 from the European Commission Joint Research Center Institute of Reference Materials. The results showed good agreement with the certificated values. The limit of detection (LOD) for each element was calculated as the standard deviation of the blank sample concentration. The LODs were 0.0016, 0.0017, 0.0017, 0.0018, 0.0017, 0.008, 0.011 and 0.009 mg/L for Pb, Ni Cd, Cr, As, Mn, Hg and Co in urine, and 0.006, 0.01, 0.006, 0.006, 0.004, 0.128, 0.001 and 0.003 mg/kg dry weight for As, Pb, Ni, Cd, Cr, Hg, Co and Mn in hair. The limits of quantification (LOQ) were defined as ten times the standard deviation. LOQs were 0.0053, 0.0057, 0.0056, 0.006, 0.005, 0.028, 0.0377 and 0.0302 mg/L for Pb, Ni Cd, Cr, As, Mn, Hg and Co in urine and 0.0216, 0.0352, 0.0233, 0.0211, 0.0149, 0.4277, 0.0244 and 0.0105 mg/kg dry weight for As, Pb, Ni, Cd, Cr, Hg, Co and Mn in hair. Metals with concentrations below the LODs were replaced with one-half of the LOD [36]. Each sample was tested in triplicate, and the relative standard deviation did not exceed 5% in any case. The laboratory-fortified blanks showed percent recoveries of As, Hg, Pb, Ni, Cd, Co, Mn, and Cr was 101(2.7), 89[4], 108(4.7), 107(3.2), 106(3.7), 107(2.5), 100(2.9) and 106(2.5) %(SD), respectively.

### Statistical analysis

SPSS software version 22 for Windows (IBM SPSS, version 22, USA) was used for demographic characteristics of participants analysis. Fisher’s exact was used to compare the demographic characteristic between the production line and back office workers. Element concentrations in urine and hair samples of printing factory workers were analyzed using GraphPad Prism version 9 software (USA, 2021). Normality of distribution of data was tested using the Shapiro–Wilk test. The data showed a non-normal distribution, and non-parametric methods were therefore applied throughout the analysis. Median, geometric means and geometric standard deviations were calculated to describe the concentration of each element in the samples. The Mann–Whitney U test was used to determine statistically significant differences between the production line and back office groups for the results from each element (significance level *p* < 0.05). The Spearman’s rank correlation test was used to indicate the correlations between two sets of data at the significance level of *p* < 0.05. A simple linear regression model was used to evaluate the association between two correlated data sets.

## Results

### General characteristics of the study participants

General characteristics of participants are summarized in Table 1. 85 of the 103 printing factory workers consented to participate in the study, giving an overall participation rate of 82.5%. The mean age of workers was 46.06 ± 10.95 years old. Eight workers (9.4%) smoked cigarettes and all of the smokers (100%) were male. 45.9% of the participants worked in the printing process. A majority of workers in the production line (76.9%) had worked in the factory for more than 5 years. All participants (100%) worked for eight hours per day and five days per week. Most workers in the production line (71%) commuted to work by motorcycle, while most workers in the back office (61%) commuted to work by personal car, 54.1% worked in a local ventilation system (closed space or back office), and 45.9% worked in a general ventilation system (outdoors). Gender was significantly different between the production line and the back office (*p* = 0.001). There was no significant difference between groups for age, smoking status, duration of employment, and work commute mode (Table 1).

**Table 1:** Demographic characteristics of participants

Characteristics	Total (*n* = 85)	Production line (*n* = 39)	Back office (*n* = 46)	*p-*value^a^
Gender, n (%)				
Male	40 (47%)	26 (65%)	14 (35%)	0.001^*^
Female	45 (53%)	13 (29%)	32 (71%)	
Age (years), mean ± SD	46.06 (± 10.95)			0.117
18–20 years	1 (1.2%)	1 (100%)	0 (0%)	
20–30 years	11 (12.9%)	6 (54.5%)	5 (45.5%)	
31–40 years	15 (17.6%)	4 (20%)	11(80%)	
41–50 years	15 (17.6%)	6 (40%)	9 (60%)	
51–60 years	43 (50.6%)	24 (53.5%)	19 (46.5%)	
**Behavior**				
Smoke cigarettes, n (%)	8 (9.4%)			0.135
Male		6 (75%)	2 (25%)	
Female		0 (0%)	0 (0%)	
**Work characteristics**				
Work department, n (%)	85 (100%)	39 (45.9%)	46 (54.1%)	
Duration of employment (years), n (%)				
< 5 years	19 (22.89%)	9 (47%)	10 (53%)	0.074
5–10 years	17 (20.48%)	7 (37%)	12 (63%)	
11–25 years	20 (24.1%)	8 (33%)	12 (67%)	
26–30 years	16 (19.28%)	12 (89%)	4 (11%)	
≥ 30 years	11 (13.25%)	3 (45%)	8 (55%)	
Work commute mode, n (%)				
By Personal car	31 (36.5%)	12 (39%)	19 (61%)	0.085
By Motorcycle	17 (20%)	12 (71%)	5 (29%)	
By Public transportation	37 (43.5%)	15 (41%)	22 (59%)	

^*^p < 0.05

^a^ Fisher’s exact test

### Element concentration in urine and hair samples of printing factory workers

Median and geometric mean were calculated to show the concentration of all elements in urine and hair samples. In the urine samples, levels of five elements were found to be above the standard values (As 78.82%, Cd 50.59%, Cr 77.65%, Ni 92.94%, and Pb 17.65%) (Table 2). The hair samples showed the corresponding levels of seven elements (Hg 18.82%, Pb 82.35%, Ni 98.82%, Cd 35.29%, Co 30.59%, Mn 61.18%, and Cr 28.24%) (Table 3). The highest rates of heavy metal contamination were from Ni (92.94% in urine and 98.82% in hair). Average concentrations of most of the metals in urine and hair were found to be above the upper reference value. The maximum concentration of all metals found in both urine and hair was much higher than the upper range of reference values (Tables 2 and 3). In urine, the values for As, Cd, Cr, Ni, and Pb were 5, 43, 236, 43, and 27 times higher than the upper range of references. In hair, the maximum concentrations detected for Hg, Pb, Ni, Cd, Co, Mn, and Cr were 10, 3, 34, 6, 312, 7, and 20 times higher than the upper range of reference values. Only a small number of workers tested positive for other heavy metals apart from Ni, but the concentration of heavy metals detected was exceptionally high. This indicates health risks in the workplace from these heavy metals.

**Table 2:** Descriptive statistical parameters for element concentration in urine of the participants (mg/L)

Element	The upper range of reference value ^a^	Min–max	Median	GM (GSD)	Positive *n* (%)
As	0.1	0.002–0.502	0.022	0.0209 (3.568)	67 (78.82)
Cd	0.000185	0.002–0.008	0.002	0.0028 (1.504)	43 (50.59)
Cr	0.00022	0.002–0.052	0.01	0.0094 (2.274)	66 (77.65)
Ni	0.003	0.002–0.128	0.018	0.0157 (2.381)	79 (92.94)
Pb	0.00067	0.002–0.018	0.004	0.0037 (1.807)	15 (17.65)

GM (GSD): geometric mean (geometric SD); n (%) out of 85 participants

^a^ Agency for Toxic Substances and Disease Registry (ATSDR), 2018

**Table 3:** Descriptive statistical parameters for element concentration in hair of the participants (mg/kg dry weight)

Element	The upper range of reference value ^b^	Min–max	Median	GM (GSD)	Positive *n* (%)
Hg	1.66	0.161–16.557	0.6866	0.8654 (3.276)	16 (18.82)
Pb	4.57	0.212–12.207	0.8098	0.9849 (2.566)	70 (82.35)
Ni	0.90	0.231–31.034	1.352	1.557 (3.022)	84 (98.82)
Cd	0.17	0.133–1.013	0.2445	0.2585 (1.558)	30 (35.29)
Co	0.14	0.480–43.678	9.431	7.165 (3.568)	26 (30.59)
Mn	0.57	0.140–3.893	0.3745	0.4453 (2.098)	52 (61.18)
Cr	0.52	0.218–10.267	1.151	1.157 (3.204)	24 (28.24)

*GM* (GSD) Geometric mean (geometric SD), *n* (%) out of 85 participants

^b^ Mikulewicz et al., 2013. [37]

**Fig. 2:**
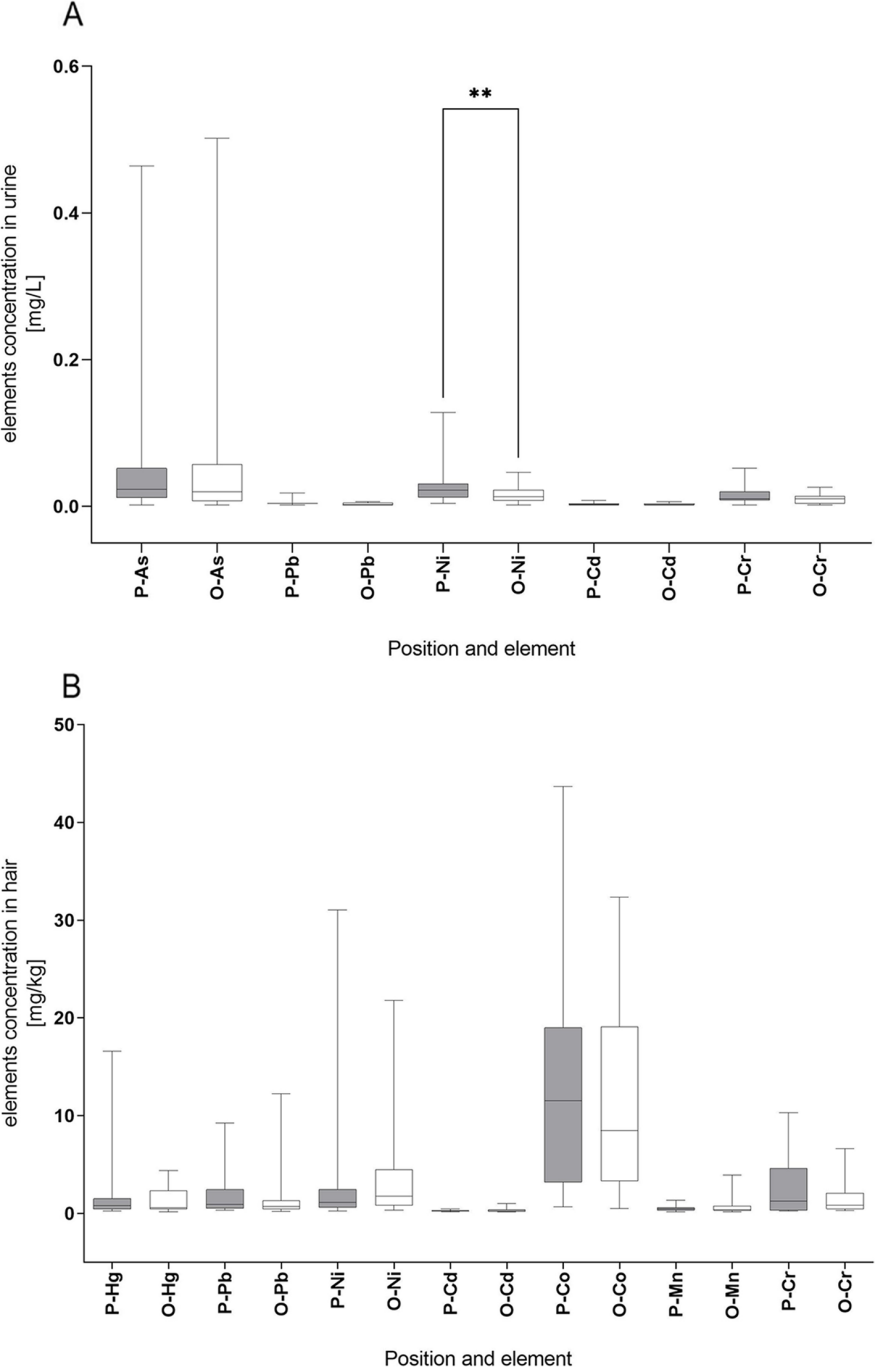
Element concentration in urine and hair samples of printing factory workers. **A** Concentrations of the selected elements in the urine of workers in the production line (P) compared to workers in the back office (O). **B** Concentrations of the selected elements in the hair of workers in the production line (P) compared to workers in the back office (O)

The participants were divided into production line and back office workers. The distribution of each element in the urine samples was compared between the two groups of participants (Fig. 2A). Only the level of Ni showed significant differences between the two groups (*p* = 0.0054), and was higher in the urine of production line workers. We also compared the concentrations of elements in hair samples between the groups (Fig. 2B). No statistically significant differences were observed.

For data that exceeded the normal range, the upper range of reference values of the element in urine and hair were used as a cut-off. The average of values that were higher than the normal range were compared for each element between the two groups. The production line workers had statistically significant higher levels of Ni in their urine than the back office workers (*p* = 0.0124). No significant changes were observed in other pairs in the data set (Table 4).

**Table 4:** Comparison element concentration between production line and back office work in urine and hair of exceeded reference value participants

Element	The upper range of reference value	Work position	Min-max	Median	GM (GSD)	*p*-value^c^
Urine (mg/L)						
As	0.1^a^	Production line	0.104-0.464 (*n*=3)	0.124	0.1815 (2.265)	>0.9999
		Back office	0.100-0.502 (*n*=3)	0.134	0.1888 (2.362)	
Cd	0.000185^a^	Production line	0.002-0.008 (*n*=23)	0.002	0.0028 (1.545)	0.7548
		Back office	0.002-0.006 (*n*=20)	0.002	0.0027 (1.468)	
Cr	0.00022^a^	Production line	0.002-0.0520 (*n*=33)	0.01	0.0112 (2.161)	0.1839
		Back office	0.002-0.026 (*n*=33)	0.01	0.0079 (2.332)	
Ni	0.003^a^	Production line	0.004-0.128 (*n*=37)	0.022	0.0218 (2.249)	0.0124*
		Back office	0.004-0.046 (*n*=40)	0.014	0.0132 (2.115)	
Pb	0.00067^a^	Production line	0.002-0.0180 (*n*=10)	0.004	0.0042 (1.842)	0.2920
		Back office	0.002-0.006 (*n*=5)	0.002	0.0029 (1.667)	
Hair (mg/kg dry weight)						
Cr	0.52^b^	Production line	1.101-10.27 (*n*=8)	2.926	2.929 (2.466)	0.3282
		Back office	0.5464-6.596 (*n*=8)	2.016	1.603 (2.239)	
Ni	0.90^b^	Production line	0.9756-31.03 (*n*=27)	2.451	3.151 (2.623)	0.2706
		Back office	0.9358-21.76 (*n*=28)	1.954	2.478 (2.334)	
Co	0.14^b^	Production line	2.926-43.68 (*n*=12)	14.71	11.25 (2.360)	0.5137
		Back office	0.4808-32.35 (*n*=12)	8.467	6.501 (3.939)	
Pb	4.57^b^	Production line	5.140-9.226 (*n*=4)	7.306	7.044 (1.318)	0.5333
		Back office	8.042-12.21 (n=2)	10.12	9.908 (1.343)	
Hg	1.66^b^	Production line	16.56 (*n*=1)	16.56	16.56 (1.000)	NC
		Back office	3.495-4.369 (*n*=2)	3.932	3.907 (1.171)	
Mn	0.57^b^	Production line	0.5917-1.339 (*n*=6)	0.9873	0.9072 (1.390)	0.2589
		Back office	0.6098-3.893 (*n*=9)	1.203	1.305 (1.950)	

*GM (GSD)* Geometric mean (geometric SD), *NC* not computable

^*^p < 0.05

^a^ Agency for Toxic Substances and Disease Registry (ATSDR), 2018

^b^ Mikulewicz et al., 2013. [37]

^c^ Mann–Whitney *U* Test

**Fig. 3:**
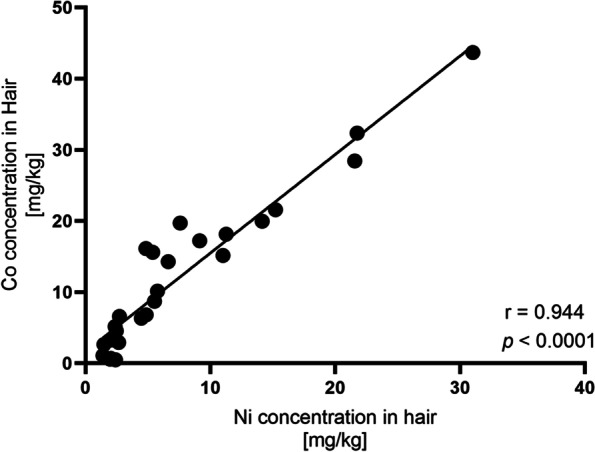
Correlation between high values of Cobalt (Co) and Nickel (Ni) concentrations in hair samples

We used the Spearman’s rank correlation test to analyze the degree of correlation between pairs of selected data. The correlation of element pairs in urine and hair samples were compared (Table 5 and 6). There was a strong, statistically significant positive correlation between high values of Co and Ni concentrations in hair samples (r = 0.944, *p* < 0.0001). The simple linear regression analysis of Co and Ni concentration in hair samples also showed a strong relationship between the two data sets (r^2^ = 0.9082) (Fig. 3). We also compared the correlation of element pairs between urine and hair samples (Table 7), but no significant changes were observed in regression coefficients compared with the overall analysis.

**Table 5:** Correlation coefficients between metal concentration in urine samples

	As	Pb	Ni	Cd	Cr
As	1	0.290085	0.32909	0.328286	0.425997
Pb		1	0.652447	0.373632	0.71951
Ni			1	0.274581	0.585974
Cd				1	0.565599
Cr					1

**Table 6:** Correlation coefficients between metal concentration in hair samples

	Cd	Cr	Ni	Co	Pb	Hg	Mn
Cd	1	-0.2028	0.19744	0.943932	0.363547	0.5	0.326057
Cr		1	0.211785	NC	0.026961	NC	-0.17895
Ni			1	0.943932****	-0.04871	0.220588	0.056103
Co				1	0.098839	0.657143	0.364743
Pb					1	-0.325	0.433385
Hg						1	-0.03571
Mn							1

*NC* Not computable (no data from same individual matched to calculate the correlation)

^****^p < 0.0001

**Table 7:** Correlation coefficients between metal concentration in the two biological samples

	Hair			
	Pb	Ni	Cd	Cr
Urine				
Pb	-0.12852	0.236503	-0.35355	-0.86603
Ni	-0.06605	0.115825	0.19744	0.211785
Cd	0.004965	-0.09217	0.250852	-0.28328
Cr	-0.02343	0.066612	0.153029	0.021259

## Discussion

Our work has established the feasibility of analyzing the internal heavy metal burden in the human body using urine and hair samples as a non-invasive approach to determine the metal contents in the urine and hair of printing workers, as well as the relationship between the metal contents and participant characteristics. As, Cd, Cr, Ni, and Pb were detected in the urine of printing workers. Hg, Pb, Ni, Cd, Co, Mn, and Cr were found in their hair. Average concentrations of most of the metals in urine and hair were found to be above the upper reference value. The workers could be separated into two groups of the production line and the back office with the significantly larger number of males in the production line group. Production line workers had much higher mean Ni levels than back-office workers. This might be due to more exposure to printed materials of the former group. There was a strong positive correlation between elevated values of Ni and Co for hair samples. The other elements did not differ significantly between production line and back office workers in either urine or hair.

We found that gender was significantly different between the production line and the back office. However, age, smoking status, duration of employment, and work commute mode were not significantly different between the production line and the back office. Most males worked in the production line and this can result in male workers being exposed to more metals than female workers.

From our findings, all smokers contained high Ni levels in their urine and hair. Our finding was consistent with several studies that found smokers' exposure to Ni through tobacco smoke was high, as were Ni levels in smokers' biological samples [38–40]. This suggests there is a need for printing workers more strict safety regulation such as wearing more protection, more frequent cleaning process, safer equipment/device, especially for males.

Both production line and back office workers had statistically different Ni levels at *p* < 0.05. The findings may be explained by different exposure to environmental sources, such as inexpensive jewelry, dyes, cosmetics, and foods [37]. The geometric mean level of Ni in the production line workers’ urine was also statistically significantly higher than that of back office workers. This result could be due to occupational exposure to nanoparticle inks in the printing factory's production process [14, 30], which is often much higher than non-occupational Ni exposure [41]. Inkjet printing uses three types of Ni: a dust of relatively insoluble Ni compounds, aerosols derived from Ni solutions (soluble Ni), and gaseous forms containing Ni (usually Ni carbonyl) [38, 42]. Ni exposure levels vary by industry, and Ni can enter the body via inhalation, skin contact, or ingestion [39]. The production process of the printing industry may release Ni dust into the air [24, 40, 43]. The toxicity of Ni dust depends on the solubility of Ni compounds. According to the United States Agency for Toxic Substances and Disease Registry, employees who are exposed to more than 10 mg Ni/m^3^ as Ni compounds develop lung and nasal sinus cancer [44].

Ni exposure can cause contact dermatitis, lung fibrosis, and alveolitis [39, 44–47]. Ni exposure increases lung and nasal cancer risk in human and animal studies as well [37, 48]. A possible mechanism could be that Ni can deplete glutathione levels, bond to the sulfhydryl groups of proteins, and induce genotoxicity, which contributes to carcinogenicity [45, 48]. The IARC classifies soluble and insoluble Ni compounds as Group 1 (carcinogen to humans), and Ni and alloys as Group 2B (possibly carcinogenic to humans) [37]. The printing industry also emits Co, according to the various studies [49–51]. Co has the potential to both benefit and harm human health. Co is a component of vitamin B12, and is useful to humans in this particular form. However, Co poisoning can cause lung, cardiac, and skin problems [52]. Animals exposed to high doses of Co have also shown liver and renal damage [46]. Workplace exposure to Co can cause fibrotic alterations in lung tissue [53, 54]. The possible mechanism is that Co nanoparticles induce oxidative stress, lung inflammation and injury, and cell proliferation, which further results in DNA damage and DNA mutation in both animal and human studies [55–57]. The IARC classifies Co in Group 2B (possibly carcinogenic to humans) [2].

We found that printing factory workers were exposed to Ni and Co, but not Pb, although this is known to be a risk in the printing industry [30, 58, 59]. These differences might be because the mean half-life of the minor fraction of Ni and Co is several years [60, 61], but the mean half-life of Pb in the human body is 30 days [62, 63].

The strength of the relationship between Ni and Co showed very strong magnitude, suggesting that Ni and Co might originate from the same source, which is the production line. A study by Mervat et al., 2014 reported considerable positive associations between levels of Ni and Co in hair samples from dental laboratory technicians [64], which was consistent with our findings. Workers in the printing industry and dentistry laboratories are exposed to several metals and metal mixtures. A study by Patel et al., 2012 demonstrated that co-exposure to Ni and Co enhances cytotoxicity and oxidative stress in human lung epithelial cells [65]. This strong inter-element correlation between Ni and Co could magnify the health effects [65] and should be further investigated.

In printing factories, workers are exposed to fumes and dust containing Ni and its compounds in printing color cartridges [66]. Similar studies examined concentrations of metal nanoparticles in printing factories and the highest levels were found in the ink preparation areas and the pressing operations [13, 21, 25, 67, 68]. In this study, the printing workers used inappropriate personal protection equipment. Workers in the production line wore gloves and masks off and on. The geometric mean level of Ni in the production line workers’ urine was statistically significantly higher than that of back office workers. This result supports occupational exposure to nanoparticle inks in the printing factory's production process. All production line workers should be more strictly protected with the same United States Environmental Protection Agency (US EPA) control measures such as measurements of the employees' air exposure levels, additional safety regulation such as wearing more protection, more frequent cleaning process, safer equipment/device regardless of males or females [69].

Numerous recent studies have reported using urine and hair analysis to determine heavy metal concentrations and signs of cell injury [10, 70, 71]. To our knowledge, there were limited occupational-related studies in metal intoxication using laboratory experiments. Our results addressed the potential application of hair and urine samples for the evaluation of metal levels in humans. These data findings increase awareness of the possible dependency and impact interactions of elemental exposure in the workplace environment.

On the other hand, this study has some limitations. We demonstrated in a single, preliminary study that printing workers came from one factory that used printing technologies. We also noted that heavy metal levels determined in this study are only measurements due to the limited sample size. Further study should be performed using a large number of clinical samples. Decision-makers should monitor workplace air contaminants, educate printing workers regarding the use of personal protective equipment in their workplaces, and establish a biological monitoring program for printing workers. Shorter work times in contaminant areas should also be scheduled.

## Conclusions

The exposure of printing factory workers to trace heavy metals presents health risks and is harmful for both production line and back-office workers with the higher average contents of the metals observed in their urine and hair samples than the upper limit values. The concentrations of Ni in the production line group with more exposure to the printed materials were found to be significantly higher than the back office group. A strong inter-element correlation between Ni and Co in hair samples which suggests that these metals might originate from the same source as well as warrant for additional investigation, especially along the production line. This study provides the insights into possible dependencies and impact interactions of heavy metal exposure in the printing industry, which could lead to the development of approach for evaluating the exposure of printing workers to metal compounds, such as an air and biological monitoring program.

## Supplementary Information

**Additional file 1:** **Fig S1.** The workflow diagram of this study.

## Data Availability

The data used and analyzed during the current study are available from the corresponding author upon request.

## Abbreviations

As: Arsenic
Cd: Cadmium
Cr: Chromium
Co: Cobalt
Pb: Lead
Mn: Manganese
Ni: Nickel
Hg: Mercury
ICP-OES: Inductively coupled plasma optical emission spectrometry
