# Supplementary figures and images for: “Assessing exposure of printing factory workers in thailand to selected heavy metals using urine and hair as non-invasive matrices”
Source: BMC Public Health. 2023 Jan 5;23:31. doi: 10.1186/s12889-022-14807-0 (PMC9817298; doi:10.1186/s12889-022-14807-0)

Supplementary material


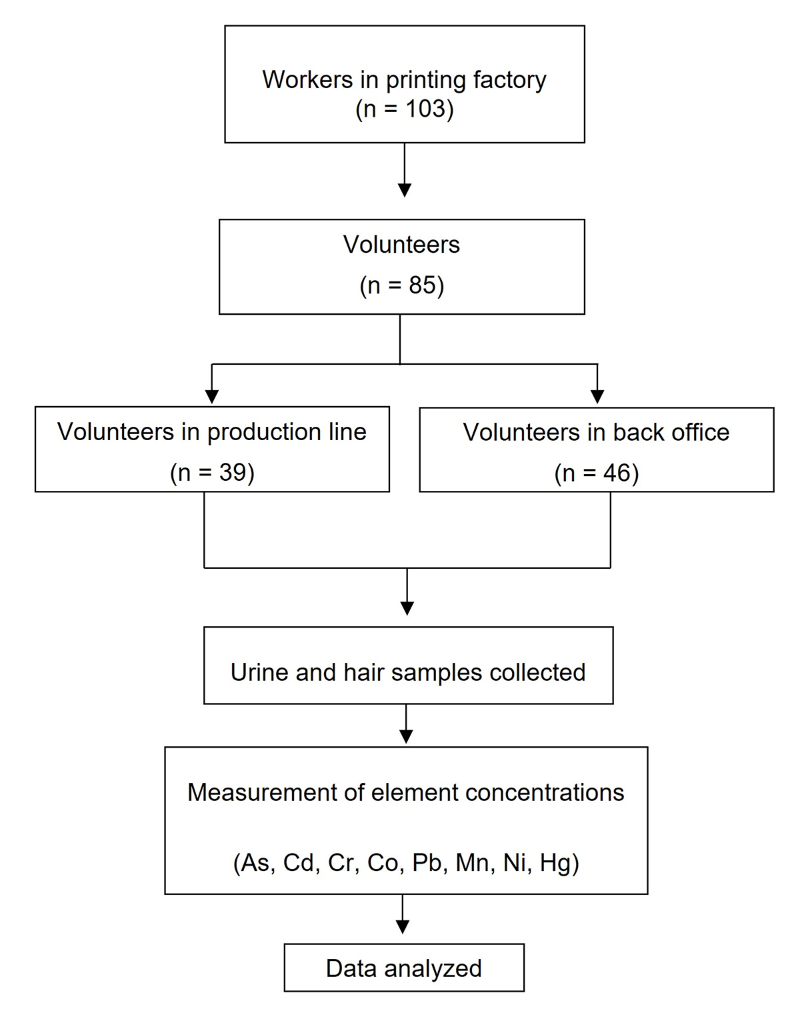


Fig S1. The workflow diagram of this study.

Supplement: Supplementary file 1 — Additional file 1: Fig S1. The workflow diagram of this study. [file 12889_2022_14807_MOESM1_ESM.docx]
